# Social brain volume is associated with in-degree social network size among older adults

**DOI:** 10.1098/rspb.2017.2708

**Published:** 2018-01-24

**Authors:** Seyul Kwak, Won-tak Joo, Yoosik Youm, Jeanyung Chey

**Affiliations:** 1 Department of Psychology, Seoul National University, Gwanak-ro 1, Gwanak-gu, Seoul, South Korea; 2 Department of Sociology, University of Wisconsin-Madison, Madison, WI, USA; 3 Department of Sociology, Yonsei University, Yonsei-ro 50, Seodaemun-gu, Seoul, South Korea

**Keywords:** social brain hypothesis, social network size, complete network, in-degree, orbitofrontal cortex, mentalizing

## Abstract

The social brain hypothesis proposes that large neocortex size evolved to support cognitively demanding social interactions. Accordingly, previous studies have observed that larger orbitofrontal and amygdala structures predict the size of an individual's social network. However, it remains uncertain how an individual's social connectedness reported by other people is associated with the social brain volume. In this study, we found that a greater in-degree network size, a measure of social ties identified by a subject's social connections rather than by the subject, significantly correlated with a larger regional volume of the orbitofrontal cortex, dorsomedial prefrontal cortex and lingual gyrus. By contrast, out-degree size, which is based on an individual's self-perceived connectedness, showed no associations. Meta-analytic reverse inference further revealed that regional volume pattern of in-degree size was specifically involved in social inference ability. These findings were possible because our dataset contained the social networks of an entire village, i.e. a global network. The results suggest that the in-degree aspect of social network size not only confirms the previously reported brain correlates of the social network but also shows an association in brain regions involved in the ability to infer other people's minds. This study provides insight into understanding how the social brain is uniquely associated with sociocentric measures derived from a global network.

## 1. Introduction

Primates have evolved to adapt to complexities of social living and their higher-order intellect is primarily suited to social problem-solving [1]. To deal with such social problems, the utilization of adaptive cognitive abilities including mind reading, tactical deception and coalition-formation are required [2,3]. According to the ‘social brain hypothesis,’ living in a large social group imposes computationally demanding information, so primates evolutionarily developed a bigger neocortex volume to adapt to such a load [4]. The ontogenetic evidence supports this conception in that complexities of the species' social environment (e.g. group size) correspond well to brain neocortex size [5]. As functioning in a highly interrelated society requires individuals to process and encode considerable social information, the maximum number and complexity of social relationships are likely to be limited by individual differences in cognitive capacity [6,7]. Thus, it is possible to predict that individuals with greater brain resources for social information processing can effectively manage larger social networks.

Recent human neuroimaging studies further support the social brain hypothesis in that regional volume and connectivity of the ‘social brain' regions are correlated with the size of social network one maintains. The social brain refers to the brain regions that consistently show activation in neuroimaging studies while engaging in a variety of social cognitive tasks. Specific regions of the social brain are typically comprised of two network systems: the amygdala network [8] and the mentalizing network [9]. Disruptions in the amygdala–orbitofrontal cortex (OFC) network result in severe social impairment possibly due to difficulties in facial emotion judgement [10], social punishment learning [11], social value processing [12] and affiliative behaviours [13] indicating its central role in social functioning. Accordingly, structural and functional characteristics of the amygdala and its highly connected OFC regions have shown the most consistent positive correlation with the individual differences in social network size [14–21]. On the other hand, brain regions involved in the ability to infer other people's minds, also known as the theory of mind (TOM), can be another neuroanatomical basis of social network size [17,18,22,23]. Mentalizing process reliably recruits activities in the dorsomedial prefrontal cortex (dmPFC), temporo-parietal junction (TPJ) and precuneus. Larger brain capacity in these regions is associated with better performance in social cognition tasks [23–25]. While the two social brain systems are anatomically and functionally distinct from each other, both systems simultaneously play an important role in perceiving value-laden social information, thus comprising the core brain regions in addressing complex social problems [22,26,27].

In the context of the social brain hypothesis, various indices including the Social Network Index [28], the Norbeck Social Support Network Score [29] and the number of friends in online social networks [18], have been used to measure the complexities of social group and to investigate whether larger social brain volume predicts greater capacity in managing one's social relationships. These indices measure social connectedness ranging from coarse online social connections to emotionally supportive and intimate relationships. However, it remains unexplained how the different properties of social network indices are associated with specific social brain structures. Moreover, although most of the studies have repeatedly reported the association between social network size in the OFC and amygdala grey matter structure across various indices [15], the regional volumes in the mentalizing network (e.g. dmPFC, TPJ and precuneus) do not show consistent association [8,15,20].

One of the challenges being raised in previous studies is the systematic bias and inaccuracy of the sociometrics solely relying upon respondent-centred methods. As social connections are not necessarily symmetric and reciprocal, using social network information derived from other individuals may provide a full picture of the interconnected nature of social relationships. When a social network index considers information beyond an individual level, it may allow an ecologically valid investigation of the neural basis of social networks [30,31]. However, none of the previous studies investigated how this directional information of social network size is specifically associated with regional brain volume in concert with the social brain hypothesis.

**Figure 1.**
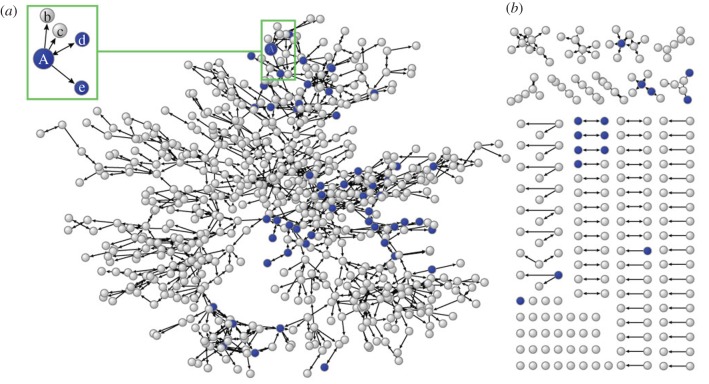
Complete network of Township K. The entire social network of community members is represented by dot-shaped nodes (*n* = 835) and directional lines (→). The subsample of MRI-scanned participants (*n* = 68) is indicated in blue. A node can have outbound ties from oneself to other nodes and inbound ties from other nodes to oneself. The largest component of 768 nodes (*a*) and the smaller or separated nodes components (*b*) are indicated.

Based on the complete mapping of the social network of an entire town, it should be possible to measure two types of individual social network size: out-degree and in-degree. The out-degree size of an individual's social network is the number of people in the whole network with whom an individual respondent most frequently or importantly interacts. The out-degree measure has been a rudimentary instrument in social network studies, not because it best quantifies the size and complexity of social networks but because it is the only measure that can be obtained from a sample via traditional survey questionnaire responses. Out-degree is generally believed to gauge the perceived amount of available social resources. On the other hand, the in-degree size of an individual social network is a measure of the number of people who cite the respondent as an important social relationship, and is obtainable through a survey that collects real names in these social networks and maps the social networks of all people in the population. Unlike the out-degree size, the in-degree size reflects the properties of popularity and aptitude of the respondent as a social tie. For example, in figure 1, individual A's out-degree network size is four because this respondent has four outbound social connections (A → b, c, d, e), whereas the in-degree network size is one because the respondent has only one inbound social connection (d → A). This approach delineates two qualitatively different directional aspects of social network size.

Meanwhile, the association between social network and brain structure may also arise from general health mechanisms, especially in the elderly population [32]. Socially integrated older adults may benefit from instrumental and emotional support via social connections. Thus, a rich social network has been suggested as one of the protective factors against adverse late-life health outcomes including cardiovascular fitness [33] and inflammatory response [34]. Although the amygdala volume showed parallel correlations among both young and older adults in a previous study [14], it is possible that brain areas contributing to a larger social network are significantly affected by age-related changes and the health benefits of social networks [35]. Based on our previous study reporting the association between self-rated health and in-degree size of an individual's social network [36], further investigation is necessary to clarify how individual differences in general health status account for brain structure in the elderly population.

The main purpose of this study is to construct the social network of an entire town, identify the directional information (i.e. in-degree and out-degree) of its social network and compare neuroanatomical correlates of two social network size indices. By examining both in-degree and out-degree types of social network size, this study purports to clarify the nature of brain correlates of social network size. We used voxel-based morphometry (VBM) analysis to quantify regional grey matter density (rGMD) and investigated how inter-individual differences in social network size have their brain structural basis [37]. More specifically, we examined whether VBM analysis results indicate specific effects within the volume of social brain regions including the amygdala network and the mentalizing network. Since the elderly population shows extensive brain volume decrease across regions, we also examined whether the identified neuroanatomical correlates are especially vulnerable to general health and ageing factors.

## 2. Material and methods

### (a) Participants

Participants in this study were a subsample from the Korean Social Life, Health and Aging Project (KSHAP). The KSHAP is a community-based cohort study that collected information on an entire population of older adults in Township K [38]. Initial recruitment in the first wave investigation identified people who were 60 years old or older and their spouses among the 1864 residents of Township K. In the follow-up recruitment in the third wave of the study, 591 participants completed a face-to-face survey and a health examination either at home or at a community centre. All measures of social network size for this study were derived from the third wave of the social network survey. Of the 591 participants who completed the survey, 194 were administered neuropsychological tests. We excluded participants with any history of neuropsychiatric or neurocognitive disorders based on a procedure reported in a previous study [39]. According to the Health Screening Exclusion Criteria [40], 58 elderly individuals were further ruled out based on one of the following exclusion criteria: psychiatric or neurological disorders, vision or hearing problems, possessing metals in the body that cannot be removed, hypertension or diabetes uncontrollable by drugs or insulin, and having a history of losing consciousness due to head trauma, infarction or stroke. Subsequently, participants who had a high probability of having a neurocognitive disorder were excluded based on neuropsychological tests (according to age and education-based norms) and semi-structured interviews. Of the 136 participants, 73 older adults volunteered for further MRI scans. Three participants were excluded due to neurological conditions identified by a radiologist, while two participants were excluded due to not completing the scans and poor image quality. The remaining 68 participants were included in the final dataset. The group included healthy older adults (42 females) who were on average 71.38 (s.d. = 6.40, range = 59–84) years old and who had an average of 6.47 (s.d. = 3.95, range = 0–20) years of formal education. The study was approved by the Institutional Review Board of Yonsei University, and all participants provided written informed consent for the research procedures.

### (b) Social network measures

Social network variables were created using the third wave of the KSHAP data. The KSHAP adopted a *name generator* from the General Social Survey and National Social Life, Health and Aging Project [38,41,42]. Individuals' social connections were constructed from a *name generator* that identifies social network members, including a spouse if any and up to five discussion partners, with information on real names, gender and residence. The questionnaire read as follows: *From time to time, most people discuss things that are important to them with others. For example, these may include good or bad things that happen to you, problems you are having, or important concerns you may have. Looking back over the last 12 months, who are the people with whom you most often discussed things that were important to you?*

To create a global (or complete) social network of Township K based on the network survey of 591 respondents, we first excluded the social ties of the respondents who were not married to any of the respondents and lived outside Township K. Then, we identified the people who appeared in different respondents' networks as identical based on the following criteria: (i) at least two out of three Korean characters in their names matched, (ii) their gender was the same, (iii) their age difference was less than 5 years and (iv) their addresses belonged to the same Ri (the smallest administration unit in South Korea). As a result, we identified a complete network of 835 township residents and 1285 social ties between them. The in-degree and out-degree social network size of an individual were defined as the number of discussion partners generated by the others or oneself, respectively. Thus, in-degree network size measured how many people cited the respondent as their discussion partner, while out-degree network size was based on how many people the respondent cited as their discussion partners. Within the total MRI participants (*n* = 68), average in-degree network size was 2.09 (s.d. = 1.81), while out-degree network size was 2.28 (s.d. = 1.62).

### (c) MRI acquisition and preprocessing

MRI images were acquired using a 3-Tesla MAGNETOM Trio 32 channel coil. Whole-brain T1-weighted images were reconstructed from 224 sagittal slices of 1 mm thickness using an MPRAGE sequence with the following parameters: TR = 2.3 s, TE = 2.3 ms, FOV = 256 × 256 mm^2^ and FA = 9°. The time between social network measurement and MRI acquisition was 16–21 months. Image preprocessing was carried out using tools implemented in Statistical Parametric Mapping software (SPM12; Wellcome Department of Imaging Neuroscience, London, UK) and executed in Matlab (MathWorks, Natick, Massachusetts). We used the New Segmentation algorithm implemented in SPM12 [43]. T1 images were bias-corrected and segmented into five tissue classes based on nonlinearly registered tissue probability maps. The East Asian International Consortium for Brain Mapping template was used for local optimization affine regularization. To spatially normalize grey matter images into a standard space with enhanced accuracy of inter-subject registration [44,45], we used Diffeomorphic Anatomical Registration Exponentiated Lie algebra (DARTEL). A customized template was created from imported versions of the grey matter tissue images. Then, the deformation field was applied to previously segmented grey matter images to implement nonlinear transformation into standardized Montreal Neurological Institute (MNI) space. During these nonlinear transformations, the total volume of grey matter was preserved with modulated images. All images were smoothed using an 8-mm full-width at half-maximum Gaussian kernel.

### (d) Voxel-based morphometry analysis

Preprocessed imaging data were analysed statistically using SPM12. Total intracranial volume (TIV) was calculated as the sum of total volumes from each segmented image of grey matter, white matter and cerebrospinal fluid. Every voxel value was proportionally scaled with TIV to adjust for total brain volume effect. Age, education, gender and the time gap between social network survey and MRI acquisition were introduced as covariates of no interest. Multiple regression models tested whether the social network measures were significantly correlated with specific voxel density. We applied multiple comparison adjustments based on FWE-corrected *p* < 0.05 with a cluster defining threshold of *p* < 0.001 (*Z* = 3.09) estimated by the Gaussian random field method implemented in SPM12 [46]. To reduce the probability of false negatives due to lack of sensitivity [47], we also opted to report clusters at a more lenient threshold of *k* > 200 considering the effect size reported in the previous studies [14,15,17]. Subjects identified as outliers in the multiple regression analysis were excluded and re-analysed to confirm the robustness of the results.

Two additional VBM analyses were conducted to examine how the late-life general health factor accounts for the relationship between the identified rGMD and social network size. First, the effect of interaction was tested in the multiple regression models by adding an age×in-degree size term and checked whether the association between in-degree size and rGMD differs across age. An attenuated age-related decrease in rGMD would be interpreted as a protective effect on brain volume reduction [48–50]. Second, we repeated VBM testing by adding terms into the regression model for possible confounding variables: depressive symptoms (30 items) [51] and self-rated health (five-point single item from ‘poor' to ‘excellent') [52]. An attenuated association between rGMD and social network size would indicate the mediating role of the added covariates.

### (e) Meta-analytic decoding with Neurosynth

To better characterize the functional implications of our VBM results, we used meta-analytic reverse inference using the Neurosynth framework (http://neurosynth.org) consisting of 11 405 fMRI studies and 3107 meta-analysed terms [53]. This method allows the identification of cognitive terms that are most probably associated with specific brain images. We uploaded the unthresholded T-map representing the GMD spatial pattern of each degree network size. The relative similarities between the cognitive terms and VBM result image were shown in a rank order of correlation. Among 50 terms of the highest correlation, methodological, redundant (e.g. mind tom, tom, TOM) and anatomical (e.g. prefrontal, medial) terms were excluded.

We also extracted the term-based reverse inference map of ‘theory mind' comprised of 140 fMRI studies (FDR *p* < 0.01, *k* > 700) and the brain regions overlapping with the thresholded social network size clusters were visually inspected. The overlapping clusters between social network size regions and Neurosynth-derived regions were identified to infer the possible social cognitive property of the VBM results.

## 3. Results

Sixty-eight elderly residents of a rural village in South Korea completed a social network survey, neuropsychological assessments and MRI scans. Both in-degree and out-degree sizes showed positively skewed distributions (in-degree skewness = 2.10, out-degree skewness = 0.77). Independent-sample *t*-tests for demographic differences between sampled participants (*n* = 68) and the rest of the social network population in the village (*n* = 523) revealed that the sampled participants had significantly more years of formal education (*t* = 2.42, *p* = 0.02) and a larger in-degree network size (*t* = 2.46, *p* = 0.02), and were younger (*t* = −2.87, *p <* 0.01). Alternatively, out-degree network size (*t* = 0.79, *p* = 0.43) and gender ratio (*χ*^2^ = 0.23, *p* = 0.70) were not significantly different. Males had marginally larger out-degree network sizes than females (*t* = −2.00, *p* = 0.05), but in-degree network size did not show a significant gender difference (*t* = −1.06, *p* = 0.30). Importantly, in-degree and out-degree sizes were not significantly correlated (*r* = 0.143, *p* = 0.25), indicating that these two indices represented distinct dimensions of an individual's social network. Neither in-degree nor out-degree network size correlated with age and years of education (*ps* = 0.24).

**Figure 2.**
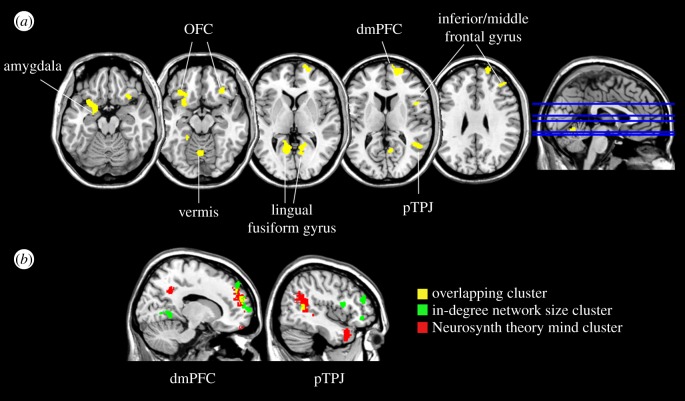
(*a*) Brain regions showing positive correlations between regional grey matter density and in-degree social network size adjusting for age, education, gender, and time gap between survey and MRI acquisition (cluster forming threshold of *p* < 0.001, uncorrected, *k* > 200). (*b*) Overlapping regions (yellow) between Neurosynth reverse inference map of ‘theory mind' (red) and clusters correlating with in-degree network size (green).

Next, multiple regression models testing the associations between rGMDs and social network size were analysed while adjusting for covariates of no interest. The results showed that the rGMD of right superior frontal gyrus (dmPFC) was positively correlated with in-degree size (*p* = 0.044, FWE corrected), while left OFC (*p* = 0.055, FWE corrected) and fusiform gyrus (*p* = 0.077, FWE corrected) showed a tendency towards positive associations (table 1 and figure 2*a*). Several regions including posterior middle temporal gyrus (pTPJ), inferior and middle frontal gyrus, and cerebellar regions also showed positive correlation with in-degree size, although they did not remain significant in the multiple comparison corrections. Importantly, no significant association with out-degree size was observed in the whole-brain analysis.

**Table 1.** Brain regions showing positive correlations between regional grey matter density and in-degree social network size adjusting for age, education, gender and time gap between survey to MRI acquisition (cluster defining threshold of *p* < 0.001, uncorrected, *k* > 200).

brain regions	peak *Z*-value	cluster size (*k*)	cluster-level FWE *p*-value	MNI coordinates		
				*X*	*Y*	*Z*
L fusiform/lingual gyrus	4.295	683	0.077	−12	−59	0
R lingual gyrus	3.932	566	0.126	17	−53	−2
R superior frontal gyrus (dmPFC)	4.258	820	0.044	18	60	26
R superior frontal gyrus (dmPFC)	3.713	243	0.524	12	51	45
L orbitofrontal cortex (OFC)/amygdala	4.132	765	0.055	−32	29	−12
R orbitofrontal cortex (OFC)	3.956	369	0.303	42	41	−6
R inferior frontal gyrus	4.131	241	0.528	44	11	14
R middle temporal gyrus (pTPJ)	4.076	216	0.585	45	−50	12
R middle frontal gyrus	4.015	400	0.264	44	41	21
cerebellar vermis	3.762	236	0.539	2	−65	−9

To test the robustness of the findings, we excluded two subjects who showed unlikely Mahalanobis distances (*D*^2^) in the multiple regression models predicting social network size (*p* < 0.05). These subjects were identified as outliers, with one showing excessively large in-degree network size (greater than ±3 s.d.). In the outlier-excluded analysis (*n* = 66), the right OFC rGMD consistently showed a robust association with in-degree network size (*Z* = 4.73, *p* = 0.032, voxel-level FWE corrected, *k* = 697). However, the dmPFC area was no longer a cluster of significant association in the FWE-corrected analysis.

Supplementary analyses were conducted to assure how age or health-related factors account for the association between in-degree size and rGMD. However, no age×in-degree size interaction effect was observed, indicating that neither of the identified rGMDs showed differential association across age. When self-rated health was introduced as a covariate, both right OFC (*Z* = 4.29, FWE corrected *p* = 0.030) and dmPFC (*Z* = 4.66, *p* = 0.027, FWE corrected) showed significant effects. The regression model including depressive symptoms also showed consistent results in left OFC/amygdala (*Z* = 4.14, *p* = 0.005, FWE corrected), right OFC (*Z* = 4.26, *p* = 0.039, FWE corrected), fusiform/lingual gyrus (*Z* = 4.23, *p* = 0.005, FWE corrected) and dmPFC (*Z* = 4.12, *p* = 0.051, FWE corrected).

To better characterize the functions implied by the regions identified with VBM analyses, we conducted meta-analytic decoding using the Neurosynth framework. The VBM results of both in-degree and out-degree social network size were decoded. When top-ranking terms were compared, the rGMD association pattern of in-degree was spatially similar to the reverse inference map of ‘theory of mind', ‘autobiographical memory' and ‘mentalizing', whereas out-degree indicated the highest similarities with ‘finger movements' and ‘motor' terms (table 2). When the meta-analysed reverse inference map of ‘theory mind' was overlapped on the thresholded VBM map of in-degree size (figure 2*a*), right dmPFC (*k* = 191) and right pTPJ (*k* = 113) showed partial overlapping clusters (figure 2*b*).

**Table 2.** Neurosynth meta-analytic decoding of the VBM results. Relative similarities between the cognitive terms and the VBM map of each social network size are noted with correlation. Among the highest 50 terms, redundant and non-cognitive terms were excluded from the list.

in-degree social network size		out-degree social network size	
term	correlation (similarity)	term	correlation (similarity)
autobiographical	0.120	movements	0.132
navigation	0.102	motor	0.111
theory mind	0.101	hand	0.105
emotion	0.100	finger movements	0.101
episodic	0.097	spatial	0.085
mentalizing	0.096	motor imagery	0.079
neutral	0.094	coordination	0.077
memories	0.090	imagery	0.077
social	0.084	finger tapping	0.073
mental states	0.081	navigation	0.073
pictures	0.079	rhythm	0.073
foot	0.078	index finger	0.069
scenes	0.076		
valence	0.076		
affective	0.074		

## 4. Discussion

Social network size was differentially associated with grey matter structure depending on the directionality of the social relationship. Social connectedness designated by other people (in-degree size) was associated with brain volumes which have been implicated in inferring social information. By contrast, self-reported social connectedness (out-degree size) did not show any association. These results provide important support for the social brain hypothesis and delineate the nature of social networks that was effective in our prediction that larger social brain volume will contribute to better management of social relationships; i.e. the in-degree size. It is noteworthy that by examining the complete social network of an entire village, we were able to measure and compare out-degree and in-degree sizes of social networks and their association with brain volumes for the first time, to our knowledge.

Out-degree directly measures the perceived amount of available social support to the respondent and shares similarities with many preceding social network indices [15]. By contrast, in-degree size measures social connections to the individual as reported by other people, indicating popularity and the capacity to attract other people for their own interest [54]. Although these two types of indices count the number of significant social relationships of real-world community dwellers, it is notable that the correlation between the two indices was statistically insignificant. Since out-degree size is based on egocentric measurement, this null result may have been due to the susceptibility to expansiveness bias of over-reporting one's actual social connections [54]. More importantly, it may not be cognitively demanding for an individual to judge and designate one's own important social ties. By contrast, in order to be designated as an important social tie (or discussion member in our survey) by others, one needs to prove that he or she remembers and understands others' various social conditions and can provide valuable social support. This can be a cognitively demanding task, especially if the person has to deal with many people of diverse backgrounds, needs and values (a large in-degree network). Consistent with the social brain hypothesis [4], the capacity to process such demanding information via the social brain would more likely to predict the number of important social connections reported by others, rather than subjective perception of social connections. Our findings suggest a fundamentally distinctive underlying mechanism of the capacity to maintain a large in-degree social network compared to a large out-degree social network.

Our result identified several social brain regions for maintaining a large in-degree social network. First, we identified the association between in-degree social network size and bilateral OFC volume. It has been suggested that this region is involved in various higher-order valuation processes [55], integrating and forming models about the value system in the environment. Individuals who successfully adapt to different social contexts may possess accurate valuation for social rewards, which makes OFC a convincing candidate for the neural substrate of adaptive social functioning. Accordingly, OFC has shown the most consistent positive association with various social network size indices in the previous review [15]. It has been shown that a larger posterior OFC volume is associated with a stronger disposition for social interaction [56], extraversion [57] and social competence [58], all of which contribute to constructing and maintaining a supportive personal social network. It is worth noting that unlike several previous studies showing the association of the ventromedial OFC with social networks [17,19], we found a positive association in the lateral portion of the OFC, which may be more relevant to memory and face processing rather than self-referential processing [59]. Consistent with previous studies, we found that the cluster of left OFC includes amygdala structure, confirming the amygdala as a hub in brain networks that support social living [8]. Lesions in the amygdala structure can cause profound social impairment and changes in social interest and affiliative behaviours, suggesting its importance in social functioning [8,10,13,60]. Among the distinctive sub-nuclei of the amygdala, it is possible that connections between the ventrolateral amygdala and OFC may play a significant role in perceiving and constructing representations of social hierarchy [61,62].

In addition, the social brain regions which are well-known neural correlates of mentalizing brain network [9,63] positively correlated with the in-degree social network size. More specifically, we identified a positive association in the right superior frontal gyrus that extends to the dorsal and medial parts of the prefrontal cortex. Along with other mentalizing network regions (e.g. precuneus, TPJ), dmPFC is a robustly activated region when encoding and inferring other people's impressions, traits, and false beliefs [24,63,64]. Accordingly, a larger brain volume or parametric activation increase in dmPFC predicts the performance of a task requiring complex levels of perspective-taking [24] or to read other people's minds through the eyes [23]. The rostral and dorsal PFC were the regions where the brain volume increased when macaque monkeys were assigned to different sizes of social groups [65]. This homologous brain volume change was also associated with the success in accommodating social rank order among these macaques, suggesting a possible link between mentalizing capacity and success in managing social relationships. On the other hand, in the exploratory analysis with a liberal threshold, we observed a positive association in another mentalizing network region. The posterior TPJ and its adjacent region in the posterior superior temporal sulcus (pSTS) are engaged in inferring other people's goals and intentions at a more perceptual level of representation [9]. Regional volume and neural activation of this area have been identified to be correlated with both social network size and performances of biological motion or gaze processing [22,25], indicating that the individual differences in personal social networks may result from basic social information processing capacity. While the amygdala–OFC network regions are involved in processing affective information of social relationships and affiliative behaviours, the mentalizing network regions appear to be mainly involved in inferences oriented toward other people's explicit behaviour [8,9]. Importantly, however, these two network systems interact with each other when processing information regarding hierarchy and popularity of real-world social network members [26,27].

It is notable that the mentalizing network regions were where previous studies failed to show a consistent effect [15,17,20] as opposed to the amygdala–OFC network. Our study suggests that when the in-degree aspect of social network size is specifically examined, the mentalizing network indeed plays a role as the social brain hypothesis would predict. The larger social brain volume involved in social perception and mentalizing is more likely to play a role in managing an individual's popularity and attractiveness (in-degree aspect), rather than subjectively perceiving the number of social connections (out-degree aspect) per se.

Another important aspect of our result is the use of meta-analytic decoding on the VBM results using Neurosynth. We found that social cognitive functions (i.e. TOM and mentalizing) strongly corresponded with in-degree VBM results, especially in dmPFC and pTPJ regions, whereas the spatial pattern of out-degree VBM map showed no relevant terms indicating social function. More specifically, the subtle GMD pattern associated with larger out-degree size does not imply social cognitive ability even without a certain statistical threshold.

Other brain regions with positive correlations with in-degree network size included the posterior and medial temporal lobe. Although these regions are not part of the major social brain networks, and they have not been representative structures involved in higher-order social inference, it is possible that the fusiform gyrus plays a role in the perception of subtle facial cues [66–68] or the encoding of the faces of socially important peer group members [27].

Meanwhile, due to the limitations of the cross-sectional design, it is possible that socially engaged individuals benefit from their larger brain volume by its general protective effect, especially in the elderly population [69]. In this study, we partly ruled out the possible effect of health status on the brain volume. The non-significant interaction (age ×in-degree) model result also implies that the identified brain association is the result of pre-existing individual differences rather than a buffered ageing effect. Nevertheless, since age-related brain atrophy in the population older than 60 s is a prominent factor that can obscure or eliminate a large amount of variance, our interpretation between brain structure and social life is still open to bidirectional interpretation and further health-related exploration using cohort longitudinal data [70,71].

One interesting finding from our elderly sample was that contrary to observations of larger social network size in elderly females compared to males in previous studies from Western elderly communities [42], elderly males showed larger out-degree social network size in our study [38]. Situated in a relatively isolated rural area in the northwest shore of South Korea, the elderly people appear to have preserved a strong patriarchal community where social activities were patriarch-centred. This may have had limiting effects on the social networking of elderly females who have catered to the needs of male spouses including social relations and activities.

We note several limitations to this study. First, the unique social and cultural characteristics of the rural village and the specific age range of the older adults may not be representative of the general population. The social networks of older adults residing in rural areas of South Korea exhibit a higher proportion of family kinship within a personal network and tend to be more cohesive than urban social networks. Additional studies in urban areas of South Korea and elsewhere are required to confirm the generality of our findings. Moreover, the range of social connections to be included was not the same as in social network indices of previous studies [15]. Specifically, we measured the size of the social network from the name generator of ‘important discussion members,' which constitutes the inner-most core within hierarchical layers of personal network. This inner core of the personal network is conceptually commensurate with ‘support clique' typically constrained within five people [4]. Further investigation of extended layers of social relationships (e.g. interacting regularly) may provide more integrated understanding of how the social network is associated with the social brain structure and function. Last, brain volume is an underspecified measure in the link between brain functioning and associated cognitive processes. The exact cellular basis of regional grey matter volume is largely unknown. The VBM method, however, generally reflects a mechanism associated with synapse density, dendritic arbour, microvasculature, and cell bodies [72,73], and such neuroanatomical characteristics sensitively detect a wide range of individual differences in behaviour and cognition [74]. We believe that individual differences in the regional grey matter can be construed as a localized abundance of neural resources and its associated cognitive capacity.

Recently, sociocentric methods have been used to quantify the patterns and structures of social relationships and have revealed that structural position within a social network can be uniquely associated with individual differences of social cognition and brain function [26,27,39,75,76] or late-life health outcome [34,77]. This approach not only effectively operationalizes sociological concepts but also characterizes how an individual gets access to social capital and gains advantage from the social network around the ego [30,31].

In summary, using social network information obtained from the complete social network of the entire village, our study has demonstrated that the two directional aspects of social connectedness, in-degree and out-degree, rely on different neural bases. The study not only confirmed the previously reported neural correlates of the amygdala network but also identified associated regions involved in mentalizing ability, extending the theoretical framework of the social brain hypothesis. It appears that individuals with small in-degree social networks might have had limiting computational capacity to manage complex social dynamics, whereas those who successfully maintained large in-degree networks were able to exercise superior social cognitive ability using their social brain capacity. This is the first study, to our knowledge, to investigate the association between two directional types of social network size and brain structure.

## Data accessibility

Data are available at https://doi.org/10.5061/dryad.7m70n [78].
